# Enhanced Toxicity Induced by Combined Exposure to Neonicotinoid Insecticides and Fluoroquinolone Antibiotics in Human Neuroblastoma SK-N-SH Cells

**DOI:** 10.3390/toxics14030195

**Published:** 2026-02-25

**Authors:** Gulijiazi Yeerkenbieke, Tao Wang, Yun Yang, Shuai Shi, Xiaoxia Lu

**Affiliations:** Ministry of Education Laboratory for Earth Surface Processes, College of Urban and Environmental Sciences, Peking University, Beijing 100871, China

**Keywords:** neonicotinoid insecticides, fluoroquinolone antibiotics, SK-N-SH cells, cytotoxicity, transcriptomics

## Abstract

Neonicotinoid insecticides and fluoroquinolone antibiotics frequently co-occur in aquatic and terrestrial environments, posing a threat to human health, yet their combined neurotoxic potential remains poorly characterized. This study aimed to assess the cytotoxicity of typical neonicotinoids and fluoroquinolones as well as their mixtures in human neuroblastoma SK-N-SH cells and identify affected pathways. SK-N-SH cells were exposed to clothianidin (CLO), imidacloprid (IMI), enrofloxacin (ENR), and ofloxacin (OFX) individually and in fixed-ratio mixtures (50% of each compound’s IC_50_) for 24 h and 48 h, and cell viability was quantified using the alamarBlue^®^ method. Single-compound dose–response testing showed time-dependent cytotoxicity, with higher potency for fluoroquinolones (24 h IC_50_: ENR 1.446 mM, OFX 2.742 mM; 48 h IC_50_: ENR 0.826 mM, OFX 2.005 mM) than neonicotinoids (24 h IC_50_: IMI 4.754 mM, CLO 5.356 mM; 48 h IC_50_: IMI 3.631 mM, CLO 4.029 mM). Concentration-addition analysis indicated that most mixtures produced synergistic interaction in reduction in cell viability, with ENR+OFX showing the strongest effect at 48 h (Observed viability 7.138% vs. Predicated viability 82.368%). RNA-seq (24 h) revealed that binary mixtures generally induced more differentially expressed genes than single exposures, and ENR-containing mixtures showed the largest transcriptomic shifts, enriching pathways related to cellular stress and injury as well as neuronal signaling and connectivity. RT-qPCR validated the changes in expressions of five key neurobiology-relevant genes (*LMO3*, *NOS1*, *ADCY8*, *FGF7* and *TNFRSF12A*). These findings highlight the importance of assessing insecticide–antibiotic mixtures when evaluating their hazards in environment.

## 1. Introduction

The growing complexity of anthropogenic chemical mixtures in the environment poses an increasingly important—and still incompletely characterized—risk to public health. The developing and adult nervous systems are especially vulnerable because of their structural complexity and limited regenerative capacity [1,2]. Unlike historical contamination episodes dominated by single compounds, contemporary exposures more often involve dynamic mixtures derived from agricultural, industrial, and household sources [3]. This reality highlights a persistent gap in conventional risk assessment, which still relies heavily on single-chemical evidence and often fails to capture interactive effects (additive, synergistic, or antagonistic) that can shape mixture toxicity [4,5]. Although the nervous system is a well-recognized target of environmental toxicants, the mechanisms by which complex mixtures elicit synergistic neurotoxicity—producing effects greater than the sum of their components—remain insufficiently resolved [6].

Among common constituents of environmental mixtures, neonicotinoid insecticides (NNIs) and fluoroquinolone antibiotics (FQs) are prominent because of their widespread use, persistence, and reported neurorelevant effects [7,8]. Imidacloprid (IMI) and clothianidin (CLO) are among the most widely used neonicotinoids globally, particularly in agricultural regions of North and South America, Asia, and Africa. Due to concerns over their toxicity to pollinators, their outdoor use has been banned in the European Union (EU) since 2018 [9]. However, in most other countries, there is no ban on the use of neonicotinoids yet. IMI is frequently detected in surface water, up to 320 μg/L in the Netherlands, with hotspots in intensive agricultural areas of Europe and North America often exceeding safety benchmarks (0.2 and 1.05 μg/L) [10,11], and a 2022 meta-analysis estimates a global mean of 119.5 ng/L [12]; IMI in groundwater was up to 1670 ng/L in Italy [13]. CLO in surface water was up to 257 ng/L in Iowa City, IA, USA [10]. Due to its high mobility, CLO poses a major groundwater threat, with concentrations reaching 3.43 μg/L in Adams, WI, USA and over 3 μg/L in Windsor, ON, Canada [13,14]. Beyond their selective insecticidal action via nicotinic acetylcholine receptors [15], NNIs have been linked to oxidative stress, mitochondrial dysfunction, and apoptosis-related signaling in neuronal systems, raising concerns for non-target organisms including humans [16,17,18]. Together, these features make NNIs well suited for interrogating how insecticide constituents contribute to neurotoxicity within realistic mixtures.

FQs, including ofloxacin (OFX) and enrofloxacin (ENR), are broad-spectrum antimicrobials used in both human and veterinary practice and are increasingly reported in aquatic environments influenced by wastewater discharge and agricultural activities [8,19]. OFX is widely detected in surface water (ng/L to µg/L), with peaks of 8770 ng/L (Europe), 10,000 ng/L (India), and 4778 ng/L (Spain). Groundwater levels of OFX reach hundreds of ng/L [20,21,22]. ENR levels in surface water range from 12 ng/L to 4.24 µg/L globally, up to 30 µg/L near livestock farms. ENR levels in groundwater in China range from 2.7 to 49 ng/L [23,24,25]. Clinically, FQs have been associated with neurological adverse reactions ranging from insomnia and dizziness to seizures [26,27]. Mechanistic evidence suggests that disruption of inhibitory neurotransmission—such as interference with the GABA_A receptor—may contribute to these effects [19], potentially converging with oxidative and excitotoxic stress responses in neural tissue [26,27]. The co-presence of NNIs and FQs therefore creates a plausible scenario for interacting neurotoxic pathways that are not captured by single-compound evaluations.

A critical but often underappreciated aspect of exposure is that NNIs and FQs can co-occur in the same environmental matrices [28]. Shared release pathways (e.g., agricultural runoff and wastewater effluent) contribute to their joint detection in surface waters and related compartments [29]. Monitoring studies in China, for example, have reported neonicotinoids (including IMI) and quinolone antibiotics (including OFX) across multiple watersheds, supporting the plausibility of combined human exposure via diet and the surrounding environment [30,31]. While individual neurotoxic mechanisms for NNIs and FQs are increasingly documented, the biological consequences of concurrent exposure remain a substantial knowledge gap. Importantly, mixtures can interact in unexpected ways, generating effects that single-chemical studies do not anticipate [4]. Although prior work in alternative models suggests potential synergistic interactions between neonicotinoid mixtures [32], systematic studies in human-relevant neuronal models are still limited.

Resolving mixture-induced neurotoxicity requires biologically relevant experimental models and pathway-level readouts. The human SK-N-SH neuroblastoma cell line is widely used in neurotoxicology and retains key neuronal characteristics suitable for probing cellular injury mechanisms induced by environmental contaminants [33,34]. Because mixture responses may involve subtle molecular perturbations preceding overt cytotoxicity, omics approaches are particularly valuable. High-throughput transcriptomics, especially RNA sequencing (RNA-seq), enables unbiased identification of genome-wide expression changes, helping to reveal dysregulated pathways and gene networks that may be missed by conventional assays and to map toxicity at a systems level [35].

This study systematically evaluates the individual and combined neurotoxic effects of representative NNIs (IMI, CLO) and FQs (OFX, ENR) in SK-N-SH cells. We hypothesize that co-exposure will produce distinct and more pronounced transcriptional disturbances than single-compound exposures, particularly in pathways governing neural function, inflammatory signaling, and cell survival. Specifically, we aim to: (1) quantify cytotoxic potency for each compound and combined mixtures; (2) profile transcriptome alterations induced by single versus binary exposures using RNA-seq; (3) identify key dysregulated pathways and gene networks related to neurodevelopment, synaptic function, and apoptosis; and (4) validate differential expression of candidate genes. This work provides mechanistic evidence to support mixture-aware neurotoxicity assessment for environmentally relevant insecticide–antibiotic co-exposures.

## 2. Materials and Methods

### 2.1. Chemicals and Cell Culture

Imidacloprid (IMI), clothianidin (CLO), ofloxacin (OFX), and enrofloxacin (ENR) of analytical grade were purchased from Beijing J&K Scientific Co., Ltd. (Beijing, China). Stock solutions were prepared in dimethyl sulfoxide (DMSO) and stored at −20 °C. The final DMSO concentration in all treatment and control media did not exceed 0.1% (*v*/*v*), a level verified to have no detectable effect on SK-N-SH cell viability. Chemical structures of the studied compounds are shown in Table A1 in the Appendix A.

The human neuroblastoma cell line SK-N-SH was obtained from Beijing BioDee Biotechnology Co., Ltd. (Beijing, China). Cells were cultured in Dulbecco’s Modified Eagle Medium (DMEM) supplemented with 10% fetal bovine serum (FBS) and 1% penicillin/streptomycin at 37 °C in a humidified incubator with 5% CO_2_. Cells were passaged at ~80–90% confluence and used for experiments within five passages.

### 2.2. Cell Viability Assay

Cell viability was measured using alamarBlue^®^ Cell Viability Reagent following the manufacturer’s protocol. Alamar Blue is a safe, stable, and water-soluble dye that is non-toxic to cells. It functions as a redox indicator, appearing as non-fluorescent indigo blue in its oxidized state and fluorescent red or pink product in its reduced state. Fluorescent signals and absorbance changes can be detected based on cellular metabolic activity. The dye was used at a concentration of 10% (*v*/*v*) in phenol red-free cell culture medium. After adding Alamar Blue, the cells were incubated for 4 h at 37 °C with 5% CO_2_ in the dark. To estimate the half-maximal inhibitory concentration (IC_50_) and its 95% confidence interval (CI) for each compound, single-compound exposure experiments were conducted. Briefly, SK-N-SH cells were seeded in 96-well plates (1 × 10^5^ cells/well) and allowed to attach for 24 h. Cells were then exposed to graded concentrations of each compound (0–8 mM for CLO and IMI; 0–5 mM for ENR and OFX) for 24 or 48 h. IC_50_ values (±95% CI) were obtained by fitting dose–response curves to a four-parameter logistic model in GraphPad Prism (version 10; GraphPad Software, San Diego, CA, USA), and goodness of fit was assessed using R^2^. For mixture experiments, cells were exposed to each compound at 0.5 × IC_50_ and to the corresponding combinations. A solvent control (0.1% DMSO) was included in each plate. Each condition was tested in 5–7 replicate wells. After exposure, alamarBlue^®^ was added to each well at 10% (*v*/*v*), and absorbance was recorded at 570 and 600 nm using a microplate reader. The calculation of cell viability is shown below:

$$Cell\;Viability=\frac{\left(A570_{treated}-A570_{blank}\right)-\left(A600_{treated}-A600_{blank}\right)}{mean\left[\left(A570_{control}-A570_{blank}\right)-\left(A600_{control}-A600_{blank}\right)\right]}\times 100$$

where A570_treated_ and A600_treated_ were absorbance readings at 570 nm and 600 nm respectively from a well containing cells exposed to the compound treatment; A570_blank_ and A600_blank_ were absorbance readings at 570 nm and 600 nm respectively from a “blank” well containing only culture medium and alamarBlue reagent (no cells); A570_control_ and A600_control_ were absorbance readings at 570 nm and 600 nm respectively from a negative control well containing untreated cells (exposed only to the vehicle), and mean referred to the averaged value.

For mixture treatments, interaction types (additive, synergistic, or antagonistic) were evaluated using the Concentration Addition (CA) model. The CA model uses the dose–response relationships of individual components to predict the expected mixture effect under simple additivity. The equation for the Predicted viability (%) of a mixture is shown below:

$$Predicated\;viability=\left[1-\sum_{i=1}^{n}\left(1-\frac{V_{i}}{100}\right)\right]\times 100$$

where *Vᵢ* was the measured (observed) cell viability under the single exposure of a compound at the corresponding concentration (%).

The predicted viability for each mixture was calculated using Microsoft Excel based on the measured (observed) cell viability values of individual compounds under single exposure conditions.

The experimentally measured cel viability (Observed viability) values were compared with the CA-predicted (Predicated viability) values, and deviations from additivity were classified as follows: additive (no significant difference between observed and predicted values), synergistic (observed values significantly lower than predicted values), or antagonistic (observed values significantly higher than predicted values) [36]. Statistical significance of deviations from additivity was assessed using a one-sample *t*-test on the difference between observed and predicted viability values (*n* = 5–7 biological replicates).

### 2.3. Transcriptome Analysis

For transcriptomic profiling, SK-N-SH cells were exposed for 24 h to each compound at 0.5 × IC_50_ and to the corresponding mixtures. For each treatment, three biological replicates were collected. Total RNA was extracted using TRIzol^®^ reagent according to the manufacturer’s instructions. RNA concentration and purity were measured using a NanoDrop 2000 spectrophotometer (Thermo Fisher Scientific, Waltham, MA, USA).

Sequencing libraries were prepared using the VAHTS Universal V6 RNA-seq Library Prep Kit for Illumina^®^ and sequenced on a NovaSeq 6000 platform (Illumina, San Diego, CA, USA) to generate 150 bp paired-end reads. Raw reads were quality-filtered and trimmed using fastp. Clean reads were aligned to the human reference genome (GRCh38) using HISAT2. Gene expression was quantified as fragments per kilobase of transcript per million mapped reads (FPKM) using StringTie (v2.0.4).

Differential expression between each treatment group and the control was analyzed using DESeq2. Differentially expressed genes (DEGs) were defined as |log_2_(fold change)| > 1 with a false discovery rate (FDR) < 0.05. Principal component analysis (PCA) based on normalized count matrices was used to visualize global transcriptomic separation among treatments.

Kyoto Encyclopedia of Genes and Genomes (KEGG) enrichment analyses and Gene Ontology (GO) were performed using the identified DEGs. Terms with Benjamini–Hochberg adjusted *p*-values (FDR) < 0.05 were considered significantly enriched. Neurobiology-related GO terms (e.g., synapse-, axon-, and neuron-associated terms) were additionally curated and visualized.

### 2.4. Quantitative Real-Time PCR Validation

To validate RNA-seq findings, five key neurobiology-related DEGs were selected for quantitative real-time PCR (qRT-PCR). Total RNA from identically treated samples was reverse-transcribed to cDNA using SPARKscript II All-in-one RT SuperMix for qPCR (SparkJade, Jinan, China). qRT-PCR was performed on a 7500 Fast Real-Time PCR System (Applied Biosystems, Thermo Fisher Scientific, Waltham, MA, USA) using 2× Universal SYBR Green qPCR Mix (SparkJade, Jinan, China) and gene-specific primers (Table 1). Thermal cycling conditions were 95 °C for 30 s, followed by 40 cycles of 95 °C for 10 s and 60 °C for 30 s, then a dissociation stage of 95 °C for 15 s, 60 °C for 60 s, 95 °C for 15 s, and 60 °C for 15 s. Relative expression was normalized to β-actin and calculated using the 2^−ΔΔCt^ method. For each treatment, three biological replicates were analyzed, with two technical replicates per sample.

### 2.5. Statistical Analysis

Data are presented as mean ± standard error of the mean (SEM), unless otherwise stated. For comparisons among multiple treatments, one-way analysis of variance (ANOVA) followed by Dunnett’s post hoc test was applied. For mixture-additivity testing, a one-sample *t*-test was used to compare the difference between measured and CA-predicted values. A two-sided *p*-value < 0.05 was considered statistically significant. Significance levels are indicated as * *p* < 0.05, ** *p* < 0.01, *** *p* < 0.001, and **** *p* < 0.0001. All statistical analyses were performed using GraphPad Prism (version 10; GraphPad Software, San Diego, CA, USA).

## 3. Results

### 3.1. Effects of Single and Combined Exposures of Neonicotinoid Insecticides and Fluoroquinolone Antibiotics on SK-N-SH Cell Viability

**Figure 1:**
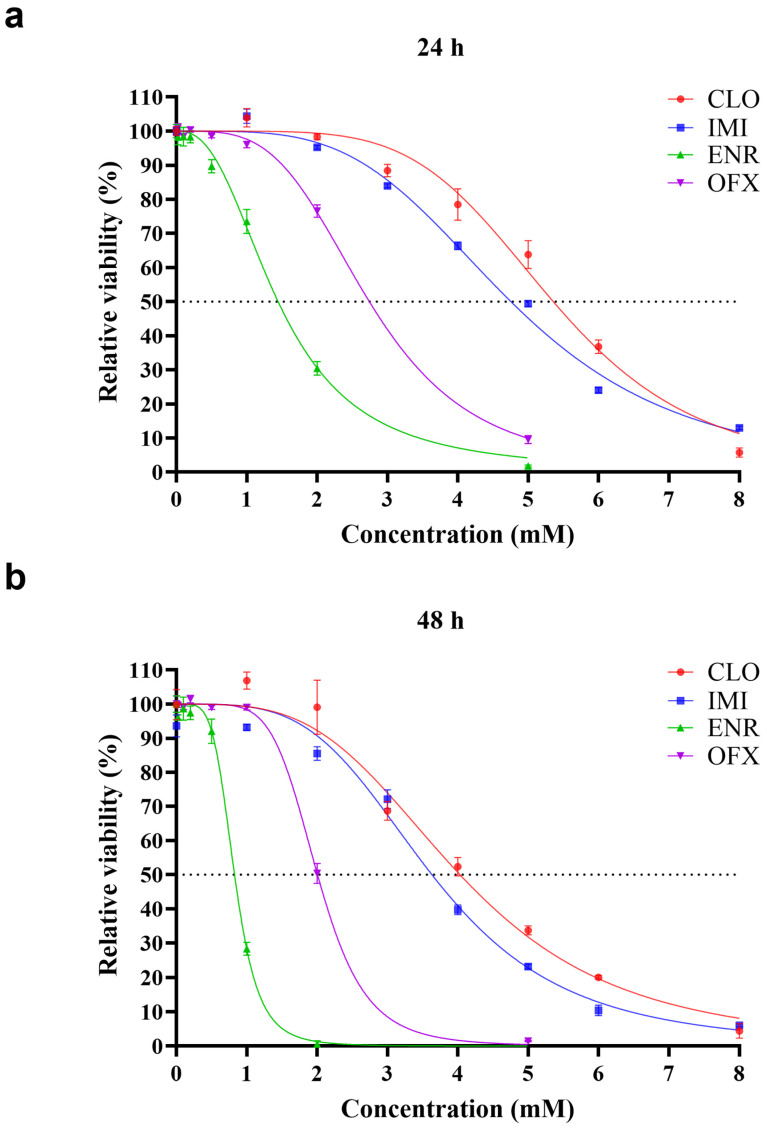
Relative viability of SK-N-SH cells after single exposure to neonicotinoid insecticides (CLO and IMI) or fluoroquinolone antibiotics (ENR and OFX) at different concentrations (the dash line indicates 50% cell viability.). (**a**) Viability after 24 h of exposure. (**b**) Viability after 48 h of exposure. Data represent mean ± SEM, *n* = 5–7.

In single-exposure assays, all four compounds reduced SK-N-SH cell viability in a concentration-dependent manner after 24 and 48 h (Figure 1). Extending exposure from 24 to 48 h further decreased viability at the same concentrations, indicating time-dependent toxicity.

IC_50_ were derived from the dose–response curves (Table 2). Across both 24- and 48-h exposures, cytotoxicity ranked ENR > OFX > IMI > CLO (lower IC_50_ indicates higher toxicity), indicating that the fluoroquinolones (ENR and OFX) were more cytotoxic than the neonicotinoids (CLO and IMI), with ENR being the most toxic and CLO the least toxic.

**Figure 2:**
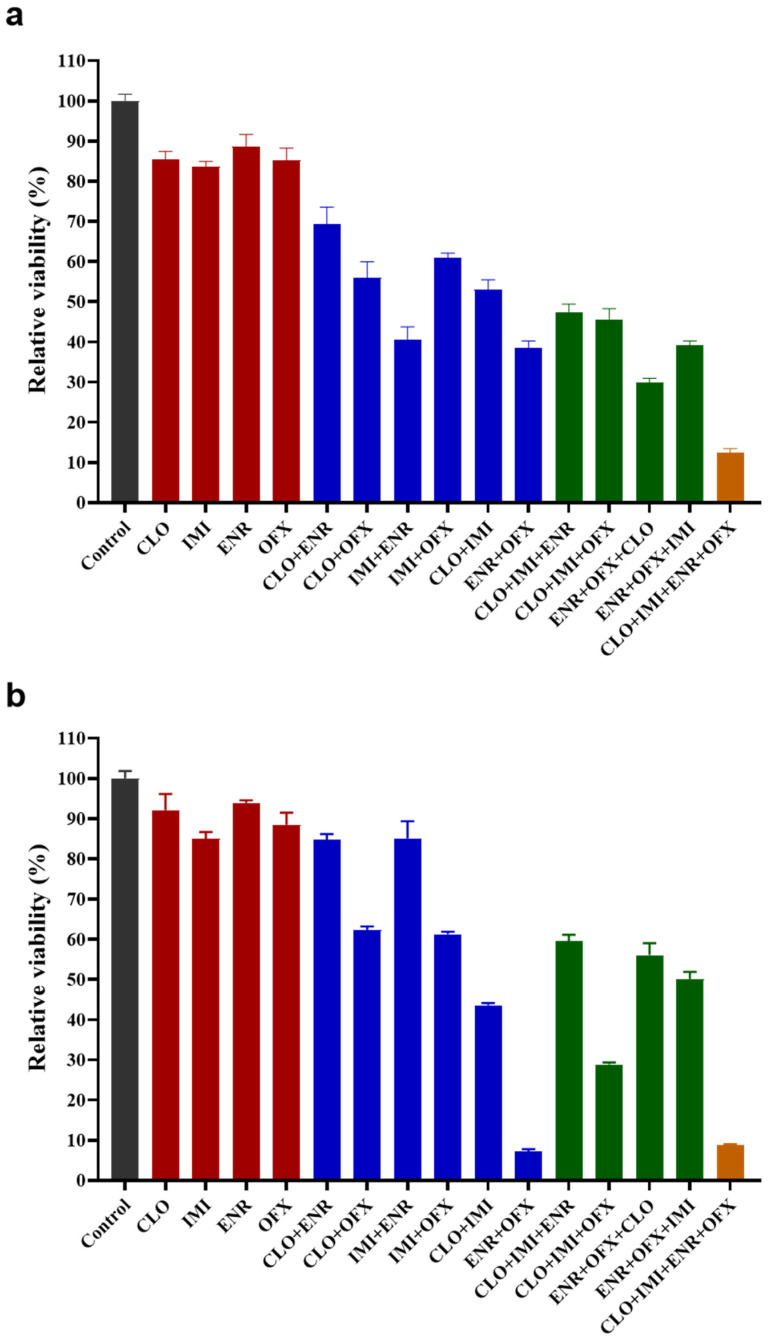
Relative viability of SK-N-SH cells following single and combined exposure to neonicotinoid insecticides and fluoroquinolone antibiotics. Cells were treated with individual compounds or co-treated at fixed concentrations (50% of the individual IC_50_ values shown in Table 2) of CLO, IMI, ENR, and OFX. (**a**) Viability after 24 h of exposure. (**b**) Viability after 48 h of exposure. Data represent mean ± SEM, *n* = 5–7.

To assess mixture interactions, SK-N-SH cells were co-exposed to two or more compounds at fixed concentrations (50% of each compound’s IC_50_). Most mixtures produced stronger inhibition of viability than the corresponding single exposures at both 24 and 48 h (Figure 2). The four-compound mixture produced the greatest reduction in viability at 24 h, while ENR+OFX mixture produced the greatest reduction in viability at 48 h (followed by the four-compound mixture).

Combined effects were quantified using the concentration addition (CA) model, and the results are shown in Table 3. CLO+ENR at both time points and IMI+ENR at 48 h showed additive effects, whereas all other mixtures were synergistic at both 24 and 48 h.

### 3.2. Effects of Single and Binary Exposures of Neonicotinoid Insecticides and Fluoroquinolone Antibiotics on the SK-N-SH Cell Transcriptome

**Figure 3:**
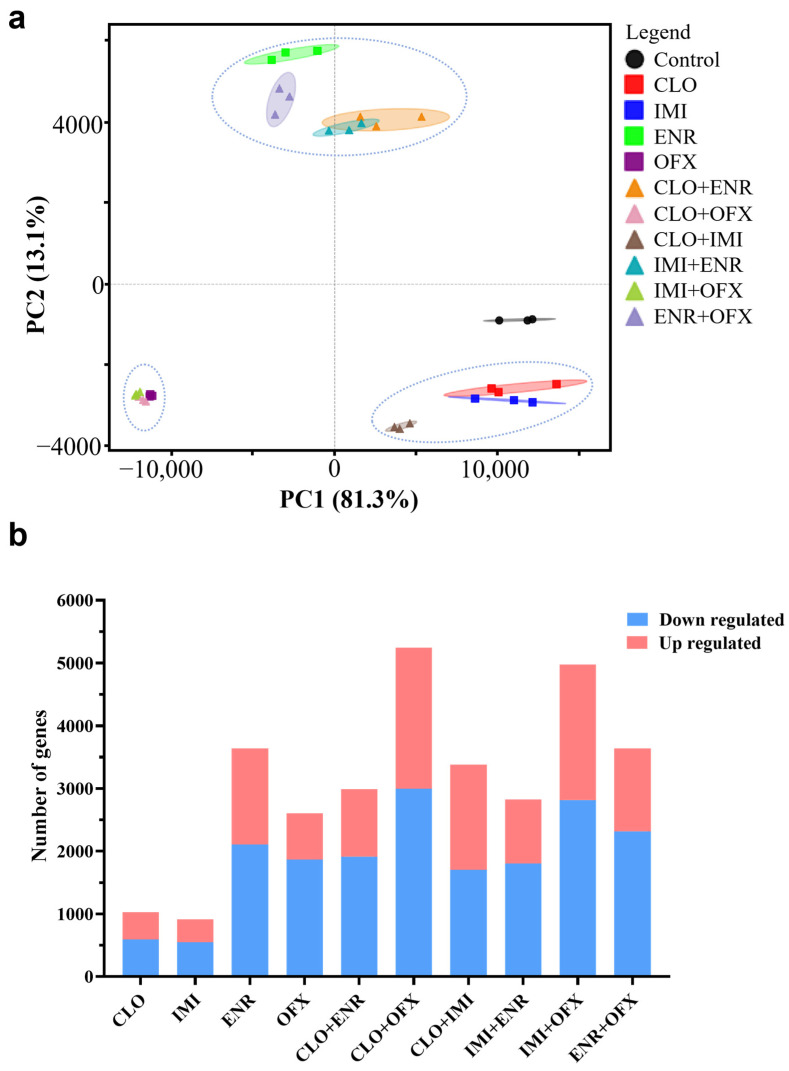
Transcriptomic profiling of SK-N-SH cells following single and combined exposures to the studied compounds. RNA-seq was performed on SK-N-SH cells treated with individual compounds or mixtures for 24 h (50% of the individual IC_50_ values shown in Table 2; *n* = 3). (**a**) Principal component analysis (PCA) plot showing sample clustering. (**b**) Number of differentially expressed genes (DEGs) identified in each treatment relative to the control.

To characterize transcriptomic responses, RNA-seq was performed on SK-N-SH cells after 24 h exposure to single compounds or binary mixtures (50% of the individual IC_50_ values shown in Table 2). Principal component analysis revealed clear separation among treatments with tight clustering of biological replicates (Figure 3a). Treatments grouped into three clusters: (1) CLO, IMI, and CLO+IMI, closest to the control; (2) OFX, CLO+OFX, and IMI+OFX; and (3) ENR, CLO+ENR, IMI+ENR, and ENR+OFX, farthest from the control. These patterns indicate that fluoroquinolones elicited stronger transcriptomic perturbations than neonicotinoids, with ENR producing the largest shift among the fluoroquinolones.

**Figure A1:**
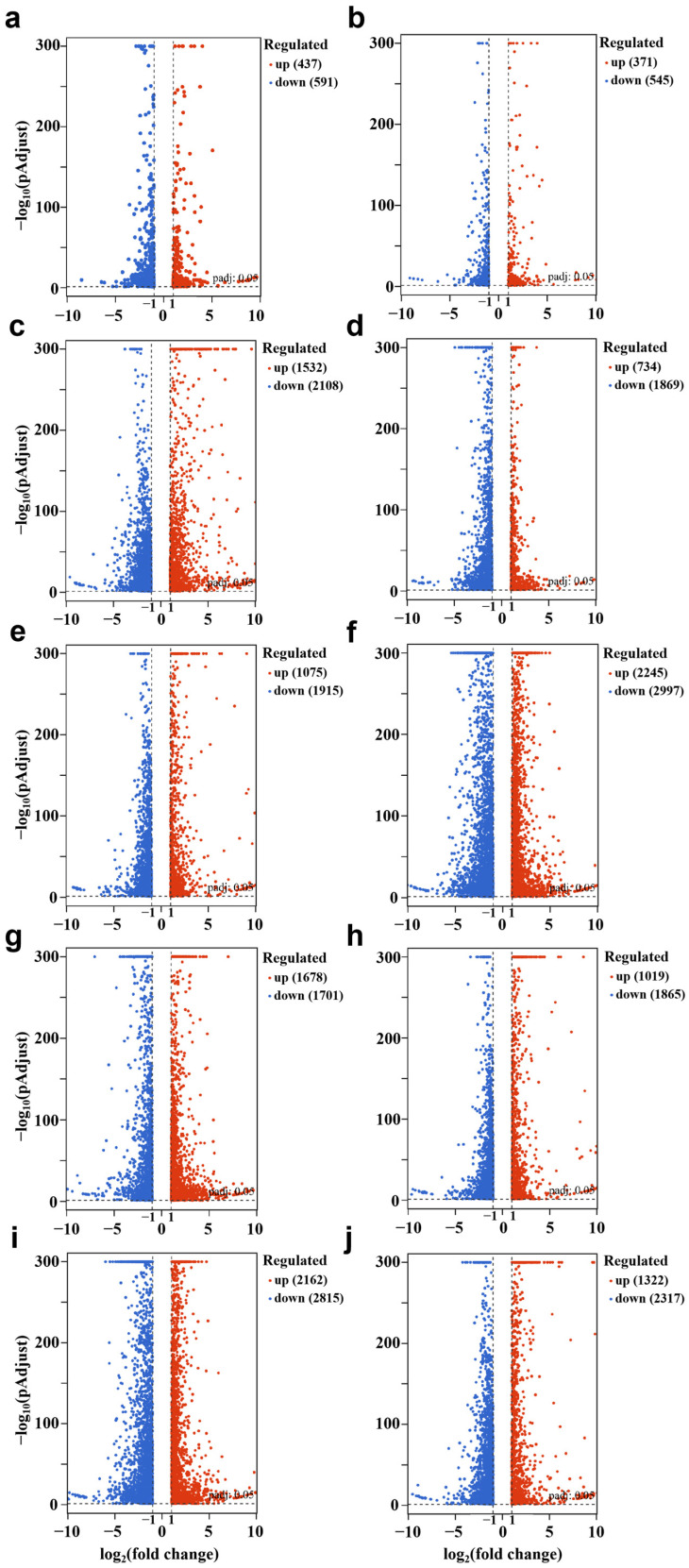
Differential expression analysis in SK-N-SH cells after exposure. RNA-seq analysis was performed on cells treated for 24 h with fixed concentrations (50% of individual IC_50_) of CLO, IMI, ENR, and OFX. *n* = 3. (**a**–**j**) Volcano plots display the results of differential expression analysis for the indicated comparison groups, vs. Control. (**a**) CLO; (**b**) IMI; (**c**) ENR; (**d**) OFX; (**e**) CLO+ENR; (**f**) CLO+OFX; (**g**) CLO+IMI; (**h**) IMI+ENR; (**i**) IMI+OFX; (**j**) ENR+OFX.

Differential expression analysis showed that binary mixtures generally induced more DEGs than the corresponding single exposures (Figure 3b). Among single compounds, ENR induced the greatest number of DEGs, followed by OFX, CLO, and IMI. Among binary mixtures, CLO+OFX induced the most DEGs, followed by IMI+OFX, ENR+OFX, CLO+IMI, CLO+ENR, and IMI+ENR. Across all conditions, downregulated DEGs outnumbered upregulated DEGs. Volcano plots summarizing DEG distributions are provided in Figure A1.

### 3.3. KEGG Pathway Enrichment Analysis

**Figure 4:**
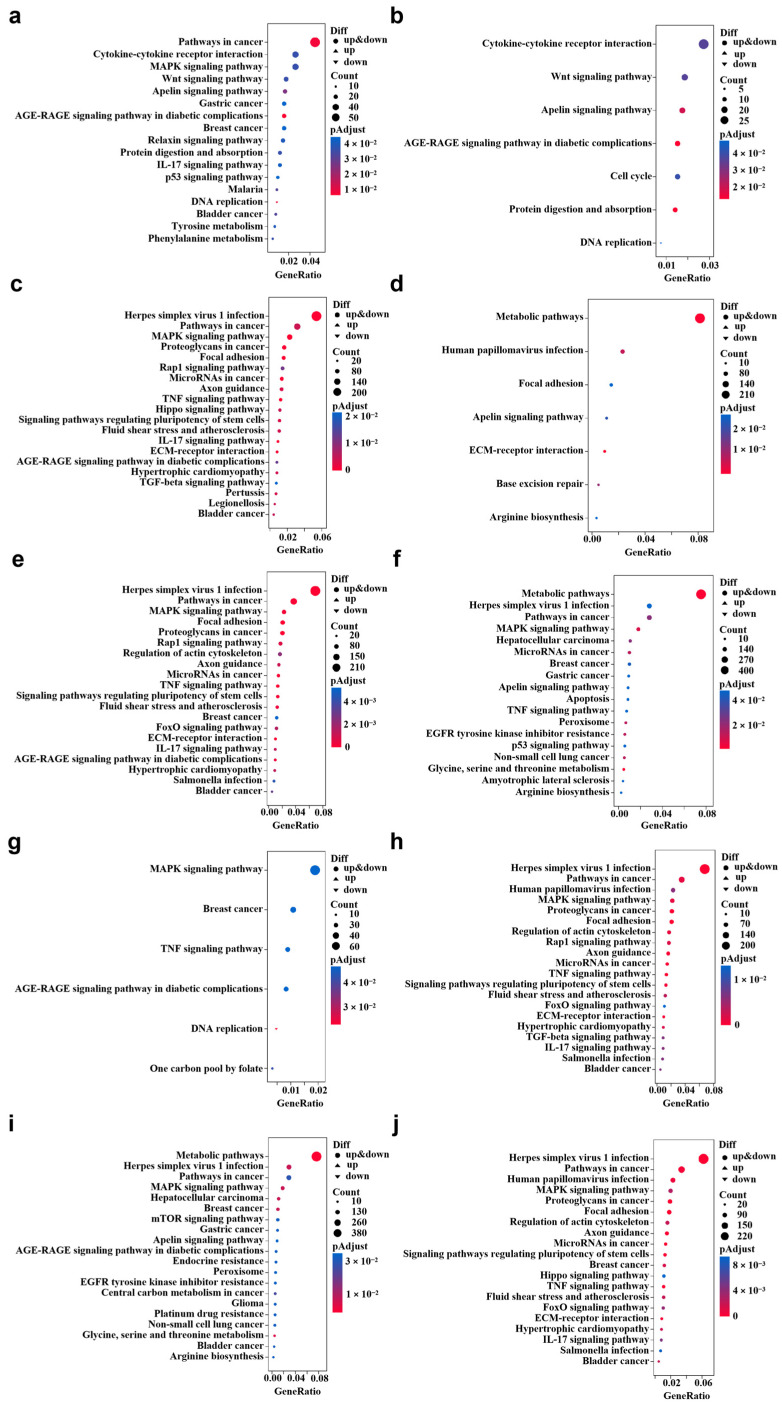
Enriched KEGG pathways under each compound treatment relative to the control. RNA-seq was performed on SK-N-SH cells treated for 24 h (50% of the individual IC_50_ values shown in Table 2; *n* = 3). (**a**) CLO; (**b**) IMI; (**c**) ENR; (**d**) OFX; (**e**) CLO+ENR; (**f**) CLO+OFX; (**g**) CLO+IMI; (**h**) IMI+ENR; (**i**) IMI+OFX; (**j**) ENR+OFX.

KEGG pathway enrichment analysis was performed using DEGs to identify biological pathways perturbed by each exposure. The numbers of enriched KEGG pathways were 17 (CLO), 7 (IMI), 23 (ENR), 7 (OFX), 34 (CLO+ENR), 18 (CLO+OFX), 6 (CLO+IMI), 29 (IMI+ENR), 29 (IMI+OFX), and 35 (ENR+OFX). With the exception of CLO+IMI, binary mixtures enriched more pathways than single compounds. ENR+OFX yielded the largest number of enriched pathways, consistent with its strong cytotoxicity. The top 20 enriched pathways for each treatment are shown as bubble plots (Figure 4a–j).

Overall, enrichment profiles showed both shared and treatment-specific signatures. Under single exposures, ENR and CLO primarily enriched pathways in Human Diseases and Environmental Information Processing; OFX mainly enriched Metabolism and Environmental Information Processing; and IMI predominantly enriched Environmental Information Processing. Across binary mixtures, enriched pathways were largely concentrated in Human Diseases and Environmental Information Processing.

Six of the seven pathways enriched by IMI were also enriched by CLO: Cytokine-cytokine receptor interaction, Wnt signaling pathway, Apelin signaling pathway, DNA replication, AGE-RAGE signaling pathway in diabetic complications, and Protein digestion and absorption. CLO additionally enriched pathways such as the p53 signaling pathway, MAPK signaling pathway, and Pathways in cancer. In the CLO+IMI mixture, additional pathways including the TNF signaling pathway and One carbon pool by folate were observed, alongside shared pathways such as DNA replication and AGE-RAGE signaling in diabetic complications.

Only two pathways were shared between OFX and ENR under single exposure (Focal adhesion and ECM-receptor interaction). ENR enriched a broader set of signaling and disease-related pathways, including Herpes simplex virus 1 infection, Pathways in cancer, Proteoglycans in cancer, MicroRNAs in cancer, MAPK signaling pathway, TNF signaling pathway, Signaling pathways regulating pluripotency of stem cells, Axon guidance, and IL-17 signaling pathway. In contrast, OFX enrichment was dominated by Metabolic pathways. Most of these pathways were also observed in binary mixtures containing ENR and/or OFX. Notably, FoxO signaling pathway and Salmonella infection were enriched in the ENR-containing mixtures (ENR+OFX, CLO+ENR, and IMI+ENR), suggesting additional pathway perturbations under co-exposure. The Apelin signaling pathway was enriched under single exposures to CLO, IMI, and OFX, and under the CLO+OFX and IMI+OFX mixtures.

### 3.4. GO Enrichment Analysis

**Figure 5:**
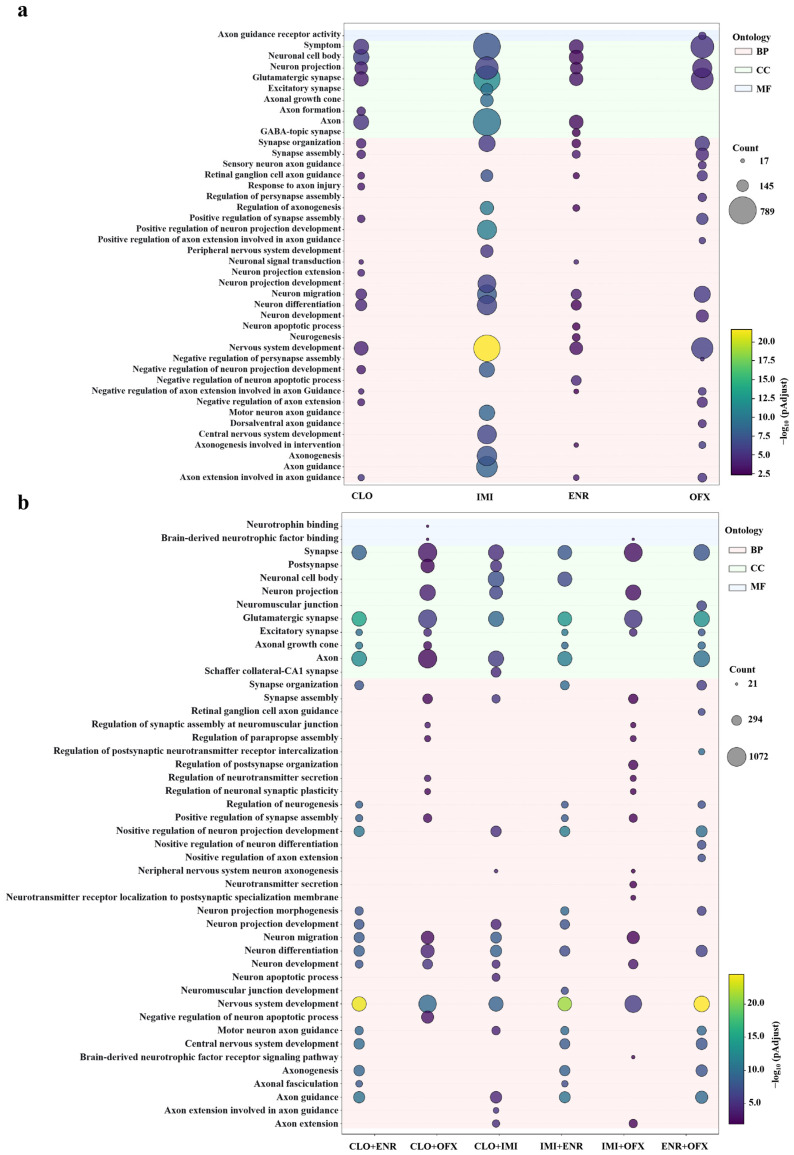
Neurobiology-related GO term enrichments under single and binary exposures. RNA-seq was performed on SK-N-SH cells treated for 24 h with compound concentrations at 50% of the individual IC_50_ values shown in Table 2. Bubble charts display the top 20 significantly enriched GO terms associated with neuronal functions and processes for each treatment versus control (*n* = 3). (**a**) Single exposures; (**b**) Binary exposures.

To evaluate neurobiology-related functions in a more focused manner, Gene Ontology (GO) enrichment analysis was performed. The top 20 significantly enriched GO terms associated with neuronal functions and processes for each treatment are shown in Figure 5. Across single exposures (CLO, IMI, ENR, and OFX), enriched terms were dominated by neural development and synaptic functions (Figure 5a). Cellular component terms highlighted synapse- and postsynapse-related structures (e.g., neuronal cell body, neuron projection, axon, axonal growth cone), while biological process terms emphasized synapse organization/assembly, axon extension, axonogenesis, and multiple neuron development processes (e.g., neuron differentiation, neuron migration, neurogenesis, nervous system development). Molecular function signals included axon guidance receptor activity. Notably, IMI displayed the broadest enrichment profile and the strongest statistical signal across multiple neurodevelopmental and synaptic terms. CLO, ENR, and OFX also showed enrichment in axon guidance and synaptic categories, although the overall signal intensity was more modest for many terms.

Binary mixtures largely recapitulated the neural and synaptic enrichment patterns observed under single exposures (Figure 5b). Across binary exposures, cellular component enrichment again concentrated on synaptic structures (synapse, postsynapse) and neuronal subcellular localization (neuron projection, axon, axonal growth cone). Biological process enrichment prominently included regulation of synapse organization, synaptic plasticity, neurogenesis, neuron projection development, and axon guidance/axon extension. Several mixtures showed strong enrichment for nervous system development and negative regulation of neuron apoptotic processes. In addition, binary exposures highlighted neurotrophin-related molecular functions that were less prominent under single exposures, including neurotrophin binding and brain-derived neurotrophic factor (BDNF) receptor binding. Together, these results suggested that binary exposures not only preserved core synaptic and axonogenesis signatures but may also emphasized neurotrophic signaling components relevant to neuronal maintenance and plasticity.

### 3.5. Validation of Key Neurobiology-Related Genes

**Figure 6:**
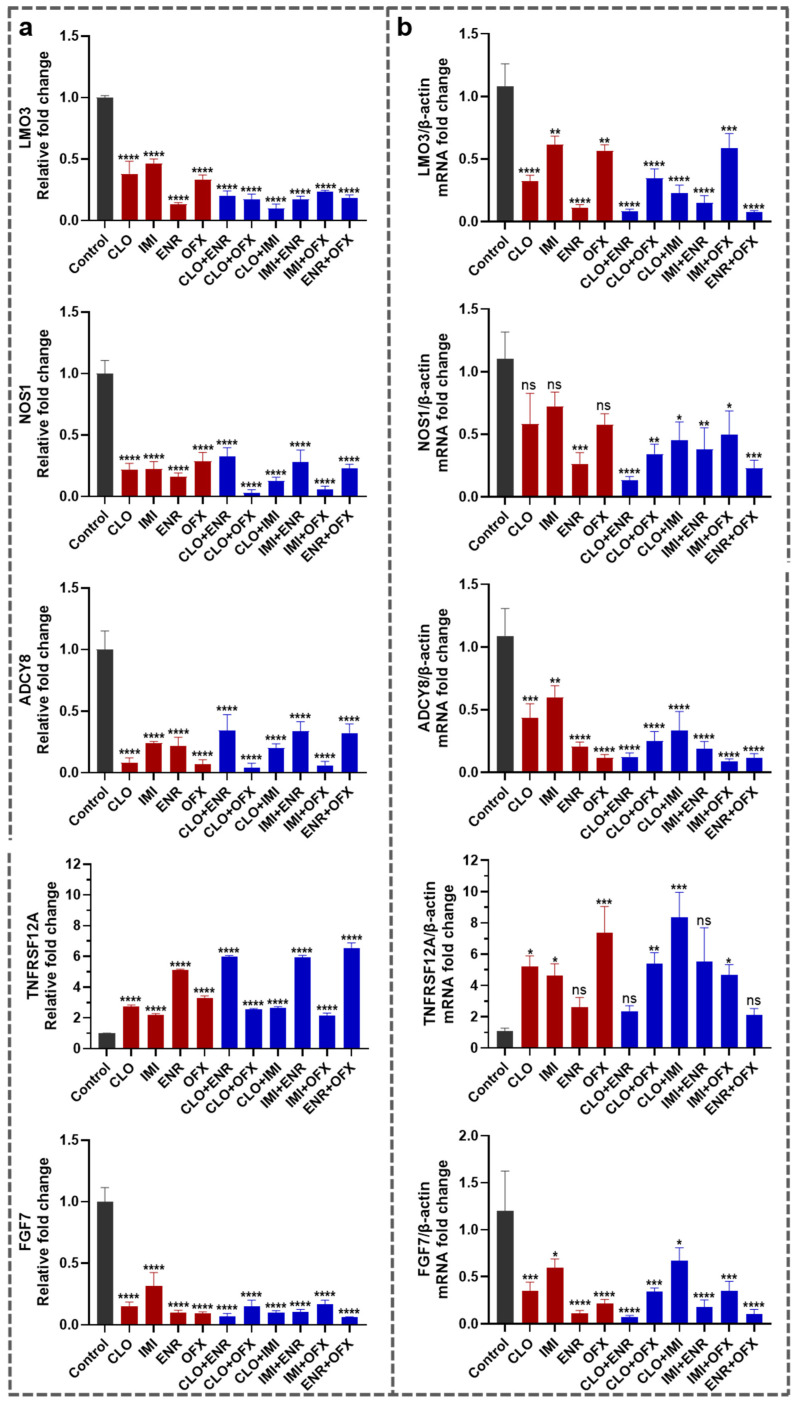
Validation of five differentially expressed genes (DEGs) in SK-N-SH cells under various treatments (compound concentrations at 50% of the IC_50_ values shown in Table 2; 24 h exposure). (**a**) Expression profiles derived from RNA-seq (relative fold change versus control). (**b**) Experimental validation by RT-qPCR (relative fold change normalized to β-actin). Data represent mean ± SEM, *n* = 3. ns *p* > 0.05, * *p* < 0.05, ** *p* < 0.01, *** *p* < 0.001, **** *p* < 0.0001, versus control.

To validate transcriptomic findings and focus on neurobiology-relevant processes, five genes related to neural development, signal transduction, and cellular stress (*LMO3*, *NOS1*, *ADCY8*, *TNFRSF12A*, and *FGF7*) were selected for RT-qPCR validation. Expression changes derived from RNA-seq and RT-qPCR are shown in Figure 6a,b, respectively. They showed consistent trends, supporting the reliability of the transcriptomic analysis. *LMO3*, *NOS1*, *ADCY8*, and *FGF7* were downregulated under various compound treatments relative to the control. *LMO3*, *NOS1*, and *FGF7* were most decreased under single ENR and ENR-containing mixtures, whereas *ADCY8* was most decreased under single OFX and OFX-containing mixtures. In contrast, *TNFRSF12A*, a gene linked to apoptotic and inflammatory responses, was upregulated under various compound treatments.

## 4. Discussion

### 4.1. Toxicity of Neonicotinoid Insecticides and Fluoroquinolone Antibiotics in SK-N-SH Cells

Using human neuroblastoma SK-N-SH cells, we found that co-exposure to neonicotinoid insecticides (clothianidin, CLO; imidacloprid, IMI) and fluoroquinolone antibiotics (enrofloxacin, ENR; ofloxacin, OFX) produced marked reductions in cell viability and frequent synergistic interactions relative to concentration-addition expectations.

In single-compound assays, all four compounds decreased viability in a concentration- and time-dependent manner, but the fluoroquinolones were consistently more toxic than the neonicotinoids across 24 and 48 h exposures (ENR > OFX > IMI > CLO). When combined at fixed ratios, most binary, ternary and quaternary mixtures yielded observed viabilities substantially lower than predicted, indicating that co-occurring agricultural contaminants can exert greater-than-additive cytotoxicity in a neuronal cell model. These findings expand mixture-toxicity concerns for neonicotinoids beyond insecticide–insecticide combinations and highlight antibiotics as potentially important co-stressors in neurotoxicity-relevant contexts.

At the cellular level, neuroblastoma models showed that neonicotinoids could reduce viability and that mixtures of neonicotinoids could display synergy at low-to-moderate concentrations [32]. Recent mechanistic work in SH-SY5Y cells indicated that IMI could induce mitochondrial dysfunction, oxidative stress, DNA damage and apoptosis at sufficiently high concentrations, supporting the plausibility of non-nAChR toxicity pathways in mammalian cells [37]. The presence of a nitro group in both IMI and CLO (Table A1) is consistent with this proposed oxidative stress mechanism, as nitro-reduction can generate reactive oxygen species. Experimental and pharmacological evidence suggested that some fluoroquinolones could lower the seizure threshold through interactions with GABAA receptor binding sites, and structure–activity analyses had identified substantial differences in excitatory potency among individual fluoroquinolones [38,39]. In our study, ENR and OFX produced lower IC_50_ values and stronger viability suppression than CLO or IMI, consistent with the broader neuroactive liability of fluoroquinolones and supporting the inclusion of these antibiotics in mixture screening when neuronal endpoints are of concern.

To quantify mixture interactions, we applied the concentration-addition (CA) model, which is widely used in mixture toxicology as a reference for non-interaction when components can be treated as effect-based dilutions of one another [40,41,42]. In our dataset, most binary and higher-order combinations produced observed viabilities significantly lower than CA predictions. CLO+ENR remained additive at both time points, and IMI+ENR approached additivity at 48 h. This non-uniformity is expected because interaction strength can vary with ratio, timing, and endpoint, and because compounds may converge on partially overlapping pathways at some conditions while diverging at others. A fixed-ratio design provides a focused test of interaction at selected ratios but does not map the full response surface. Future work using response-surface designs and complementary reference models would help generalize the interaction landscape and identify ratios that maximize synergistic risk.

### 4.2. Transcriptomic Signatures Under Exposures to Neonicotinoid Insecticides and Fluoroquinolone Antibiotics in SK-N-SH Cells

RNA-seq at 24 h revealed clear separation of treatment groups by principal component analysis, with ENR and ENR-containing mixtures producing the largest global shifts in gene expression. The observation that mixtures generally yielded more differentially expressed genes and enriched more KEGG pathways than single compounds was in agreement with the viability data, suggesting that combined exposures broadened the set of perturbed cellular programs rather than simply intensified a single pathway. Enrichment of signaling pathways related to cellular stress and inflammation (e.g., MAPK, p53, TNF/IL-17 signaling, cytokine–cytokine receptor interaction, and apoptosis) was consistent with a scenario in which combined exposure accelerated injury signaling and limited recovery, thereby contributing to synergistic loss of viability. A salient discovery emerged from the ENR-containing exposures: a robust enrichment of the “Herpes simplex virus 1 infection” pathway. While seemingly anomalous, this pathway is a recognized orchestrator of innate immune and pro-apoptotic signaling within cells [43,44]. Its activation here implies that ENR may be instigating a sterile, virus-mimetic state of inflammation in neural cells—a novel mechanistic insight that aligns with emerging theories linking similar inflammatory states to chemical-induced neurotoxicity and neurodegenerative processes [45].

GO enrichment further emphasized neurobiology-relevant processes such as axonogenesis/axon guidance, neuron projection development, synapse organization and synaptic plasticity. Neurotrophin signaling is a central regulator of neuronal differentiation, connectivity and synaptic function, and disruption of neurotrophin pathways can alter synapse maturation and plasticity [46,47]. The repeated appearance of synaptic and axonal terms across single and mixture exposures suggested that, beyond acute cytotoxicity, these compounds might perturb neuronal connectivity-related gene networks in this cell model. That mixtures often recapitulated or strengthened these enrichments supported the idea that combined exposures could reinforce neurodevelopmental or synaptic vulnerabilities through convergent or complementary pathway perturbations.

RT-qPCR validation corroborated RNA-seq trends for five neurobiology-relevant genes (*LMO3*, *NOS1*, *ADCY8*, *FGF7* and *TNFRSF12A*). Downregulation of *NOS1* and *ADCY8* was notable because nitric oxide and cAMP acted as key second messengers in synaptic signaling and plasticity. *NOS1*-derived nitric oxide could function as a retrograde messenger modulating presynaptic release and synaptic homeostasis, whereas calcium-stimulated adenylyl cyclases (including *ADCY8*) contributed to cAMP signaling that supported activity-dependent synaptic modification [48,49]. Reduced *FGF7* expression was also consistent with altered synaptic balance, as *FGF7* (alongside other FGFs) promoted inhibitory presynaptic terminal differentiation during development [50]. Conversely, upregulation of *TNFRSF12A* (Fn14) suggested engagement of injury-responsive signaling, as the TWEAK/Fn14 axis could be induced in the neurovascular unit and had been linked to neuronal death and neuroinflammatory processes [51]. Although these validated changes did not establish causality for cytotoxicity, they provided prioritized targets for follow-up studies using pathway perturbation and functional readouts.

### 4.3. Environmental Implication, Limitations and Future Directions

Neonicotinoids and fluoroquinolones can plausibly co-occur in agricultural and peri-urban settings where insecticides are used for crop protection and antibiotics are applied in animal production. Our observation that ENR, OFX, CLO and IMI mixtures mostly exhibited synergy in a neuronal cell model suggested that insecticide-antibiotic combinations warrant inclusion in mixture screening efforts, particularly for endpoints related to neuronal viability, oxidative stress and synaptic signaling.

This study has several limitations. First, SK-N-SH cells are a practical human-derived model for neurotoxicity screening, but they do not fully represent mature neuronal networks, glial contributions, or blood–brain barrier transport as such, the current findings cannot be directly extrapolated to in vivo neurotoxicity. Second, alamarBlue^®^/resazurin reduction is an integrated readout that depends on cellular redox capacity and metabolic activity; it is sensitive and convenient but does not specify whether injury proceeds via apoptosis, necrosis or other regulated programs [52]; Third, mixtures were evaluated at fixed ratios, which was informative for targeted hypotheses but did not capture ratio-dependent interaction landscapes; Fourth, only the parent compounds were evaluated, without considering the toxicity of the metabolites, which may not fully explore the mechanism. Finally, the tested concentrations might exceed typical environmental levels, so extrapolation to population exposure requires careful contextualization, including toxicokinetic considerations and chronic low-dose paradigms.

Directions for future studies include: (i) assess lower and more environmentally realistic concentrations over longer exposure durations; (ii) integrate mechanistic endpoints (ROS generation, mitochondrial membrane potential, ATP depletion, DNA damage, caspase activation, and neurite outgrowth) to connect pathway perturbations to phenotypic outcomes; (iii) test additional neural cell systems (e.g., differentiated SH-SY5Y, neuron–glia co-cultures) to probe cell-type specificity; and (iv) evaluate interaction surfaces using multiple reference models. These steps would improve mechanistic inference and strengthen the translational relevance of mixture findings for environmental and human health risk assessment.

## 5. Conclusions

Neonicotinoid insecticides clothianidin (CLO) and imidacloprid (IMI) as well as fluoroquinolone antibiotics enrofloxacin (ENR) and ofloxacin (OFX) reduced viability of human neuroblastoma SK-N-SH cells in a dose- and time-dependent manner, with fluoroquinolones exhibiting higher cytotoxic potency (ENR > OFX > IMI > CLO based on 24 h and 48 h IC_50_s). Fixed-ratio mixture testing indicated that combined exposures often deviated from concentration additivity, with several binary and higher-order mixtures producing greater-than-additive (synergistic) loss of viability, particularly ENR+OFX at 48 h. Transcriptomics further showed that ENR-containing mixtures elicited the strongest gene-expression disruption and enriched pathways linked to cellular stress/injury (e.g., MAPK signaling, FoxO signaling) alongside neuronal signaling and connectivity processes (e.g., axon guidance and neurotrophin-related terms). RT-qPCR validation corroborated RNA-seq trends for five neurobiology-relevant genes (*LMO3*, *NOS1*, *ADCY8*, *FGF7* and *TNFRSF12A*). Overall, these results highlight the importance of mixture-aware hazard characterization for co-occurring insecticides and antibiotics. Future work should evaluate environmentally relevant concentrations, longer and repeated exposures, and more physiologically representative neuronal models with functional neurotoxicity endpoints.

## Figures and Tables

**Table 1:** Primers used for qRT-PCR.

Gene	Forward Primer	Reverse Primer
*β-Actin*	5′-TGTAACCAACTGGGACGATATGG-3′	5′-GATCTTGATCTTCATGGTGCTAGG-3′
*LMO3*	5′-TCCACCCTGTACACTAAAGCTAAT-3′	5′-AATTTGTCTCCAACACAAAATCTCT-3′
*NOS1*	5′-TCTCCTCCTACTCTGACTCC-3′	5′-TTGTGGACATTGGATAGACC-3′
*ADCY8*	5′-CATCGCTATGAGAACGTCAGTA-3′	5′-AGTCCCCCAGGATTTTAATACG-3′
*FGF7*	5′-GCAACTGAACTTACTACGAACT-3′	5′-ATTGACCTCTTCCTATCTGTGA-3′

**Table 2:** IC_50_ of SK-N-SH cell viability under single exposure to the studied compounds.

Compound	24 h			48 h		
	IC_50_ (mM)	95% CI (mM)	R^2^	IC_50_ (mM)	95% CI (mM)	R^2^
CLO	5.356	5.192~5.521	0.969	4.029	3.581~4.208	0.963
IMI	4.754	4.660~4.850	0.989	3.631	3.502~3.757	0.978
ENR	1.446	1.361~1.535	0.980	0.826	0.787~0.869	0.988
OFX	2.742	2.637~2.852	0.990	2.005	1.960~2.056	0.991

**Table 3:** Interactions in combined exposures.

Exposure Time	Combined Exposure	Observed Viability (%)	Predicted Viability (%)	Interaction *	*p* Value
24 h	CLO+ENR	69.334	74.030	Additive	0.228
	CLO+OFX	55.997	70.645	Synergistic	<0.001
	IMI+ENR	40.630	72.312	Synergistic	<0.001
	IMI+OFX	60.906	68.928	Synergistic	0.014
	CLO+IMI	53.087	69.088	Synergistic	<0.001
	ENR+OFX	38.430	73.869	Synergistic	<0.001
	CLO+IMI+ENR	47.299	57.715	Synergistic	0.003
	CLO+IMI+OFX	45.674	54.331	Synergistic	0.012
	ENR+OFX+CLO	29.968	59.272	Synergistic	<0.001
	ENR+OFX+IMI	39.141	57.554	Synergistic	<0.001
	ENR+OFX+CLO+IMI	12.401	54.000	Synergistic	<0.001
48 h	CLO+ENR	84.677	85.997	Additive	0.736
	CLO+OFX	62.381	80.529	Synergistic	<0.001
	IMI+ENR	85.054	78.920	Additive	0.058
	IMI+OFX	61.307	73.453	Synergistic	<0.001
	CLO+IMI	43.628	78.920	Synergistic	<0.001
	ENR+OFX	7.138	82.368	Synergistic	<0.001
	CLO+IMI+ENR	59.566	78.256	Synergistic	0.002
	CLO+IMI+OFX	28.910	72.789	Synergistic	<0.001
	ENR+OFX+CLO	56.082	74.930	Synergistic	<0.001
	ENR+OFX+IMI	50.131	67.854	Synergistic	<0.001
	ENR+OFX+CLO+IMI	8.763	65.051	Synergistic	<0.001

* Additive: *p* > 0.05; synergistic: observed viability < predicted viability, *p* < 0.05.

## Data Availability

The original contributions presented in this study are included in the article. Further inquiries can be directed to the corresponding author.

## Appendix A

**Table A1:** Chemical structures of the studied compounds.

Compound	Clothianidin (CLO)	Imidacloprid (IMI)	Enrofloxacin (ENR)	Ofloxacin (OFX)
Chemical structure				
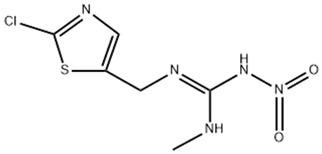
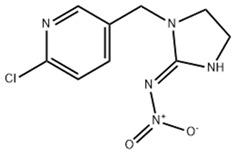
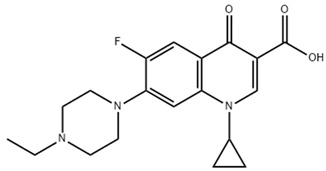
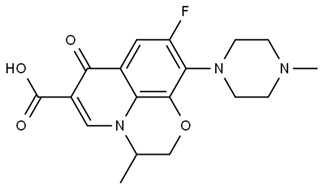
Molecular formula	C_6_H_8_ClN_5_O_2_S	C_9_H_10_ClN_5_O_2_	C_19_H_22_FN_3_O_3_	C_18_H_20_FN_3_O_4_
Molecular weight	249.67	255.66	359.40	361.37
